# Primary Sjögren’s syndrome misdiagnosed as Mikulicz’s disease: a case report

**DOI:** 10.1186/s12886-023-03090-1

**Published:** 2023-07-27

**Authors:** Tingting Ren, Rui Liu, Jing Li, Jianmin Ma

**Affiliations:** Beijing Institute of Ophthalmology, Beijing Tongren Hospital, Beijing Tongren Eye Center, Capital Medical University, Beijing, 100730 China

**Keywords:** Sjögren’s Syndrome, Mikulicz’s disease, Differential diagnosis

## Abstract

**Background:**

Sjögren’s Syndrome (SS) is an inflammatory autoimmune disease, and Mikulicz’s disease (MD) is a lymphoproliferative disorder. Both MD and SS are more common in middle-aged female, and the dry eyes could be presented in both of them with different degree. The MD patients are characterized by symmetrical swelling of the lacrimal glands which also can occur in the early stage of SS. And the imaging findings between early stage of SS and MD are lack of specificity. Therefore, SS and MD have similarities in the clinical manifestations, imaging and pathological findings and are confused in diagnosis.

**Case presentation:**

A 51-year-old female patient presented with bilateral swelling of the upper eyelids for 2 years. She also reported having dry mouth and dry eyes which could be tolerated. The Schirmer’s test result is positive and the laboratory tests indicate serum total IgG increased. In the bilateral lacrimal gland area could palpate soft masses. The orbital magnetic resonance imaging (MRI) examination showed bilateral lacrimal gland prolapse. While the histopathological result was considered as MD. The immunohistochemical (IHC) staining results were positive for IgG and negative for IgG4. To clarify the diagnosis, SS-related laboratory tests were performed: anti-SSA antibody (+++), anti-SSB antibody (+++), anti-Ro-52 antibody (+++). With a comprehensive consideration, the final diagnosis was SS.

**Conclusion:**

When the clinical manifestations are atypical, it is necessary to pay attention to the differential diagnosis of SS and MD.

## Background

Sjögren’s Syndrome (SS) is a chronic inflammatory autoimmune disease, the typical histopathological feature of which is the infiltration of lymphocytes in exocrine glands (especially in lacrimal glands and salivary glands), leading to clinical manifestations such as dry mouth and dry eyes [1, 2]. Xerostomia, dry eyes, fatigue and arthralgia can occur in more than 80% of the patients and are usually the reason for patient’s first visit. Glandular enlargement and various extraglandular manifestations are other presenting features [3]. Mikulicz’s disease (MD) is a lymphoproliferative disorder characterized by persistent, painless, symmetrical swelling of the lacrimal and salivary glands. The histopathological features include massive lymphocytic and plasmacytic infiltration with fibrosis, sclerosis, elevated serum IgG4 levels (> 135 mg/dl) and infiltration of IgG4-positive plasma cells (percentage of IgG4 + cells to IgG + cells > 50%) [4, 5]. SS and MD are similar in clinical features and imaging findings, and clinicians are prone to misdiagnosis. This article reports a case of Sjögren’s Syndrome with bilateral lacrimal gland enlargement that was misdiagnosed as Mikulicz’s disease, which provides references for clinical diagnosis and treatment.

## Case report

**Fig. 1:**
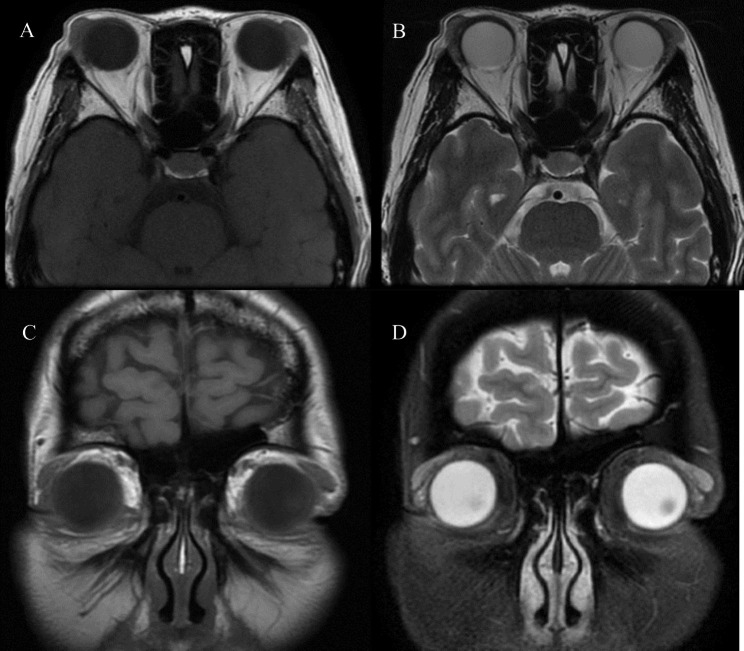
MR images of the orbit. **A**. T1-weighted images (T1WIs) of the orbital transection showing bilateral lacrimal glands moved forward and no abnormalities in the eyelids, extraocular muscles, nerves, or other tissues. **B**. Transverse orbital T2WIs showing bilateral lacrimal glands moved forward. **C**. Coronal T1WIs of the orbits showing bilateral lacrimal glands prolapse. **D**. Enhanced orbital scan showing bilateral lacrimal glands prolapse

A 51-year-old female patient presented with bilateral swelling of the upper eyelids for 2 years, which worsened in the past six months. She also reported having dry mouth and dry eyes which could be tolerated. The Schirmer’s test showed that the right eye was 2 mm/5min, and the left eye was 0 mm/5min. Ophthalmic examination showed: visual acuity—right eye, 1.0; left eye, 1.0; and intraocular pressure—right eye, 14 mmHg; left eye, 15 mmHg. Performances in the anterior and posterior segments were normal. Soft masses could be palpated in the bilateral lacrimal gland area. And the laboratory tests indicate positive syphilis antibodies, serum total IgE, IgG, IgA, light chain K and light chain L increased. Orbital magnetic resonance imaging (MRI) examination showed bilateral lacrimal gland prolapse, and no abnormalities in the eyelids, extraocular muscles, and nerves (Fig. 1). The preoperative diagnosis was bilateral lacrimal gland prolapse, but according to the clinical manifestations of dry eyes and dry mouth and the results of Schirmer’s test, the possibility of SS cannot be ruled out.

### Pathologic findings

**Fig. 2:**
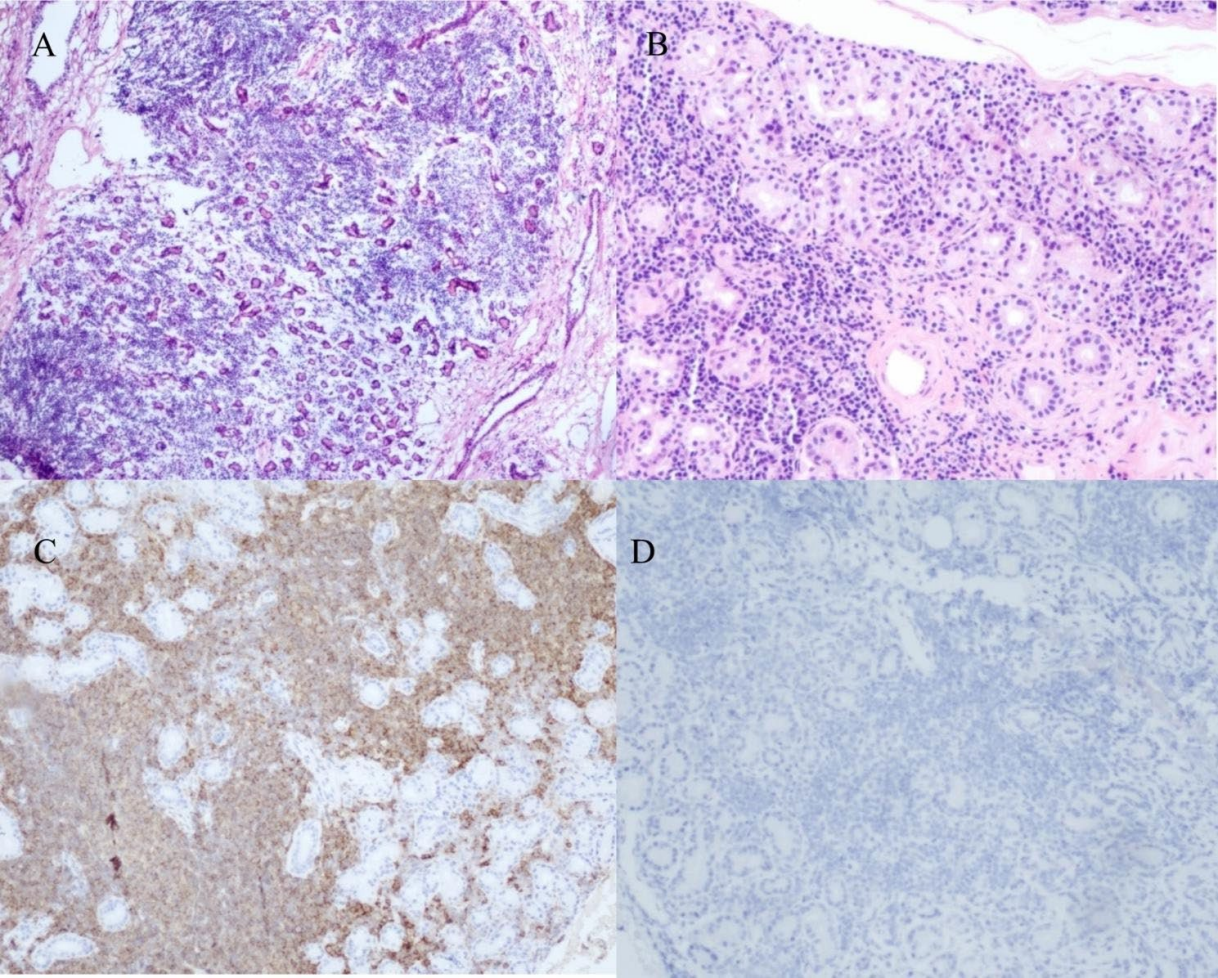
Pathological results and immunohistochemical staining of lacrimal gland lesions. **A**. Lymphocytes infiltration and lacrimal gland atrophy in the lesion (×100). **B**. Lymphocytes and plasma cells infiltration and atrophy of the lacrimal gland acini in the lesion (×200). **C**. Immunohistochemical staining for CD20 showed non-diffuse positivity of B cells. **D**. IgG4 expression was negative

In order to confirm the diagnosis, the patient underwent repositioning of the right and left lacrimal glands under general anesthesia. During the operation, the lacrimal glands were soft and slightly enlarged, a small part of the lacrimal glands was removed for histopathological examination. The results showed obvious atrophy of the lacrimal gland acinus, hyperplasia of the lacrimal duct epithelium, and infiltration of lymphocytes and plasma cells in the tissue, which was considered as MD (Fig. 2). Immunohistochemical (IHC) staining results were positive for Bcl-2, CD3, CD20, CD21, CD38, CK, Ki67, IgG, and negative for IgG4. In view of the similarity in the pathological manifestations of MD and SS, and the negative expression of IgG4 by immunohistochemical staining in this case, combined with the specific clinical features and tear secretion test results of the patient, the possibility of diagnosis of SS cannot be ruled out. In order to further clarify the diagnosis, we performed SS-related laboratory tests: anti-SSA antibody (+++), anti-SSB antibody (+++), anti-Ro-52 antibody (+++). According to the new primary SS classification criteria (ACR-EULAR) [6] in 2016, the final diagnosis was SS. After the operation, the patient was treated with oral methylprednisolone with an initial dosage of 24 mg per day for 1 week and a taper over 6 weeks. The patient recovered well in a follow-up examination a year later, and no recurrence was found.

## Discussion and conclusions

SS is one of the most common rheumatic diseases with a prevalence of about 0.5% [7]. The disease is more common in middle-aged women, with a female-to-male predominance of 10:1. The peak age of onset is 55–64 years old for female and 65–74 years old for male patients. The median age of MD is 45 years old, with a female-to-male ratio of 4:1 [8]. Both MD and SS are mainly female-onset, but the average onset age of MD is lower than that of SS. 5-10% of SS patients may eventually develop into B-cell lymphoma [9–11], while > 10% of MD patients may develop malignant lymphoma [12]. Malignant transformation can occur in both MD and SS patients, and is more common in MD.

The vast majority of SS patients have moderate to severe dry eyes, while a small percentage of MD patients may experience mild dry eyes. Lacrimal gland atrophy is seen in most SS patients, which is different from MD. The volume of lacrimal glands in SS patients can increase, decrease, or be normal at different stages of the disease process. Lacrimal gland enlargement often occurs in the early stage of the disease and is generally mildly enlarged. Therefore, for patients with normal or slightly enlarged lacrimal gland, the diagnosis of SS should be highly suspected if IgG4 is negative.

The early imaging findings of SS are lacrimal gland swelling caused by active inflammation, which is similar to MD and is easy to be misdiagnosed. In the advanced stages of the disease, cystic changes can be found due to the destruction of the lobules and fibrotic and fatty infiltration of the gland parenchyma, which presents as a “honeycomb” or “salt and pepper” appearance on MRI, and there may be punctate calcifications on computed tomography (CT) [13, 14]. Patients with a longer course of disease may present with glandular atrophy [15]. MRI in patients with MD can reveal bilateral diffusely enlarged lacrimal glands with clear borders, with low-to-equal homogeneous signal intensity distribution on T1WI and T2WI, and is obviously enhanced after enhancement. And there is no calcification within lesions on CT [16]. Therefore, Late SS shows heterogeneous signal on imaging, and gland atrophy occurs, with calcification on CT, which is easy to distinguish from MD.

The histopathology of SS and MD are similar and easy to confused. SS is characterized by periductal lymphocytic infiltration and acinar atrophy or severe destruction, while MD is characterized by non-periductal lymphocytic infiltration and acinar destruction. The frequency of apoptosis in MD is lower than that in SS. Germinal centers can appear in both, but the frequency, number and size of which in SS are lower than those in MD [17]. Studies have shown that 90% of MD have elevated serum IgG4 levels and IgG4+/IgG + ratio of tissue plasma cells, while SS patients have no IgG4 + cells [8]. In this case, the IgG4 level was not high, considering that it may be non-IgG4-elevating MD. Acinar cells in SS patients can be stained by APO2.7, and strongly stained by Fas and Fas-L, indicating the destruction of acinar cells, while APO2.7, Fas and Fas-L staining in MD patients are all negative [17].

Serological testing and minor salivary gland biopsy are currently the most reliable means of diagnosing SS [18]. Anti-nuclear antibodies (ANA) and rheumatoid factor (RF) are the most common autoantibodies in SS, and Anti-SSA/SSB antibodies positive could appear in 2/3 of SS patients, while in patients with MD or lacrimal gland prolapse (LGP) they are mostly negative. Serum IgG4 levels are elevated in the vast majority of MD, but normal in the patients with SS or LGP [4, 19].

At present, there is no effective treatment for SS. Alternative and symptomatic treatments are mainly used. The effect of glucocorticoid treatment on MD is significant, while SS does not respond to hormone therapy or the symptoms are partially improved. Both MD and SS can recur or become malignant after treatment [4, 19].

Through this case, we realize that when the clinical manifestations are atypical, it is necessary to pay attention to the identification of SS and MD. IgG4 expression in SS is usually negative, while most of MD are positive. For patients with negative serological and immunohistochemical IgG4, even if the pathology is considered as MD, the diagnosis cannot be fully confirmed. Necessary symptom consultation is required, past history, physical examination and laboratory tests (anti-SSA/SSB) should be considered comprehensively. Most SS patients have positive anti-SSA/SSB antibodies, while MD is negative, and a small number of SS patients can be negative for specific antibodies. If necessary, consult the rheumatology and immunology department.

## Data Availability

Not applicable.

## Abbreviations

SS: Sjögren’s Syndrome
MD: Mikulicz’s disease
MRI: magnetic resonance imaging
IHC: Immunohistochemical
CT: computed tomography
ANA: Anti-nuclear antibodies
RF: rheumatoid factor
LGP: lacrimal gland prolapse
